# Drug information sources in professional work—a questionnaire study on physicians’ usage and preferences (the drug information study)

**DOI:** 10.1007/s00228-023-03494-4

**Published:** 2023-04-14

**Authors:** Pia Seidel, Bo Rolander, Anna L. Eriksson, Ulf Lindahl, Susanna M. Wallerstedt, Staffan Hägg, Anders Kling

**Affiliations:** 1 Department of Integrative Medical Biology, Clinical Pharmacology and Pharmacology, Umeå University, Umeå, Sweden; 2 Futurum-Academy for Healthcare, Region Jönköping County, Jönköping, Sweden; 3 Department of Behavioral Science and Social Work, School of Health Sciences, Jönköping University, Jönköping, Sweden; 4 Department of Internal Medicine and Clinical Nutrition, University of Gothenburg, Gothenburg, Sweden; 5 Department of Clinical Pharmacology, Sahlgrenska University Hospital, Gothenburg, Sweden; 6 Region Västernorrland, Härnösand, Sweden; 7 Department of Pharmacology, Sahlgrenska Academy, University of Gothenburg, Gothenburg, Sweden; 8 HTA-Centrum, Sahlgrenska University Hospital, Gothenburg, Sweden; 9 Department of Medical and Health Sciences, Linköping University, Linköping, Sweden; 10 Department of Clinical Science, Child and Adolescent Psychiatry, Umeå University, Umeå, Sweden

**Keywords:** Drug information, Information seeking behavior, Physicians, Primary health care

## Abstract

**Purpose:**

This study aimed to explore physicians’ use of drug information in professional work, with special focus on those working in primary care, and also in relation to personal characteristics of physicians.

**Methods:**

A web-based questionnaire was distributed by e-mail to physicians in five regions in Sweden. The questions concerned drug-related queries at issue when searching for information, sources used, and factors of importance for the choice of source, as well as responder characteristics.

**Results:**

A total of 3254 (85%) out of 3814 responding physicians stated that they searched for drug information every week. For physicians working in primary health care, the corresponding number was 585 (96%). The most common drug-related issues searched for by 76% of physicians every week concerned pharmacotherapeutic aspects (e.g., dosing), followed by adverse drug reactions (63%). For 3349 (88%) physicians, credibility was the most important factor for the choice of sources of drug information, followed by easy access online (*n* = 3127, 82%). Further analyses among physicians in primary care showed that some personal characteristics, like seniority, sex, and country of education, as well as research experience, were associated with usage and preferences of drug information sources.

**Conclusions:**

This study confirms that physicians often use drug information sources in professional work, in particular those who work in primary health care. Credibility and easy access are key factors for usage. Among physicians in primary care, personal factors influenced the choice of drug information sources.

**Supplementary Information:**

The online version contains supplementary material available at 10.1007/s00228-023-03494-4.

## Background

Prescribing medicines shall be based on scientific knowledge and proven experience. To facilitate the process of prescribing, numerous sources can be searched for drug information. Indeed, a systematic review found that treatment was the first-ranked physician clinical information needed in most articles [1]. A literature review 3 decades ago suggested that commercial sources (direct mail, journal advertising, and detailing) had declined significantly in importance as sources of pharmaceutical information, whereas colleagues and conferences/conventions had increased [2]. Since then, over the last 3 decades, physicians’ usage of health information has changed dramatically. Online health information has also become part of daily work, necessitating digital skills. As information sources vary over time, revisiting the use of drug information sources in current health care is warranted. As physicians in primary care treat patients over all disciplines, knowledge about information sources used in that context could be of particular interest [3].

Information seeking behavior of physicians seems to depend on a variety of factors. Although many studies have focused on technical and organizational factors [1], there are a few studies implying that personal factors could have an impact on information seeking behaviors [2, 4]. Therefore, there is a need for knowledge on the usage of drug information among physicians, especially in relation to their personal characteristics. The aim of this descriptive study was to investigate physicians’ use of drug information in professional work, with special focus on those working in primary care. A second purpose was to explore a possible association between personal characteristics of primary care physicians and their choices of sources of drug information.

## Methods

### Study population

**Fig. 1:**
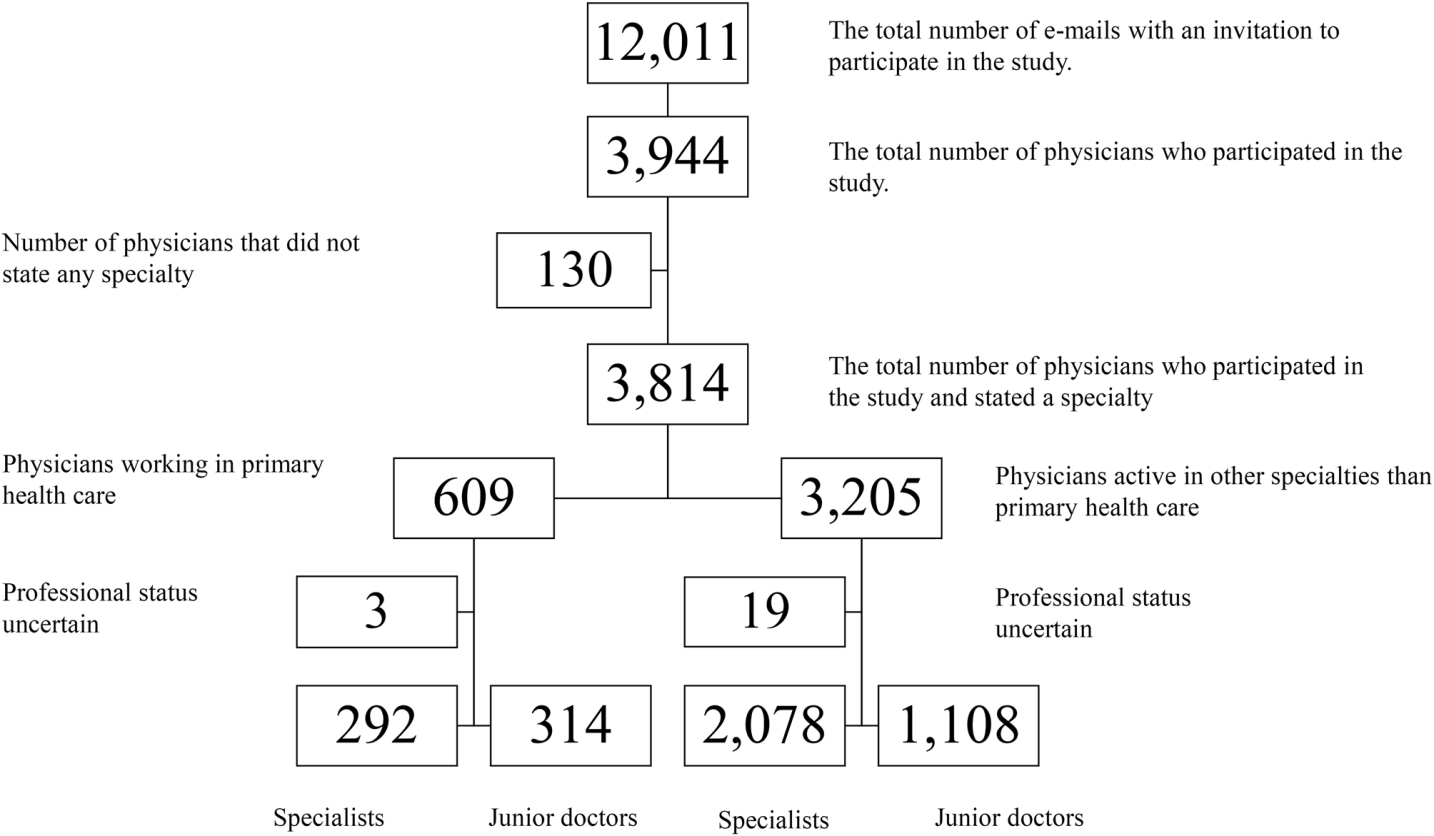
Flowchart showing numbers of responders

The study involved physicians active in five Swedish regions (Region Västerbotten, Region Västernorrland, Region Östergötland, Region Jönköping County, and Region Västra Götaland). A link to a web-based anonymous questionnaire was distributed by e-mail to all physicians with an available e-mail address (*n* = 12,011) in the study regions. E-mail addresses were obtained from the regional administrative units. The response rate was 32.8% (*n* = 3944). Of the participating physicians, 130 did not state any medical specialty for their place of work and were excluded from further analysis due to uncertainty if they were active. Consequently, 3814 physicians active in a total of 66 different medical specialties were included in the study. In addition, the largest individual specialty, physicians working in primary health care (*n* = 609), which constituted 14% of the whole study population, was analyzed separately with respect to possible associations with responder characteristics (Fig. 1).

### Questionnaire and collection of data

The questionnaire contained two sections, concerning (i) drug-related queries and type of drugs at issue when searching for information, sources used, and factors of importance for the choice of source, and (ii) responder characteristics, including age, sex, position, specialty, and extent of patient work. Predefined answer alternatives were provided as well as free text options. For some questions, the respondents could select multiple answers. It was not mandatory to respond to all questions. For validation purposes, the questionnaire was piloted in a group of physicians in a primary health care center in Umeå. A web-based tool for surveys and analysis was used to distribute the questionnaires (esMaker version NX2, Entergate AB, Sweden). In all, eleven reminders were sent. The recipients could actively choose not to get more reminders by entering a code in the e-mail.

### Statistics

All statistical analyses were conducted using SPSS version 25 (IBM Corp., Armonk, NY, USA). The dichotomized variables are presented in numbers (*n*) and percentages (%). The group of physicians active in other specialties than primary care was a very heterogeneous group, since some medical specialties use medical drugs to a greater extent, e.g., geriatrics, and others to a much lesser extent, e.g., hand surgery. With that in regard, no statistical comparisons were performed between the whole group of physicians and physicians active in primary care. To test possible associations between responder characteristics and preferences of drug information sources, we focused on physicians active in primary care. Pearson chi-square test was used, and Bonferroni correction was applied to adjust for multiple comparisons. Statistical significance was set at *α* = 0.05 (two sided).

### Ethics

The Swedish Ethical Review Authority determined that the Ethics Review Act was not applicable to this study and had no objections to the performance of the study (ref 2016/157–31). The respondents of the questionnaire stayed anonymous by automatic coding of the questionnaires within esMaker. Thus, no one, including the researchers, could link participants’ responses with their identity.

## Results

Characteristics of the responding physicians are shown in Table 1. A total of 3254 (85%) out of 3814 respondents in the study reported searching for information about drugs at least every week. In the subgroup of physicians working in primary care, the corresponding figure was 585 (96%). For physicians 50 years and younger, compared with those above 50 years of age, the proportions were 89% versus 76% (*p* < 0.001). The most common drug-related issues searched for by 2888 (76%) physicians every week concerned pharmacotherapeutic aspects (e.g., dosing), followed by adverse drug reactions searched for by 2384 (63%) physicians (Table 2). The various types of drug-related issues were encountered in the same mutual order for both groups of physicians. A total of 558 (15%) respondents chose to list at least one drug-related issue in free text, most commonly regarding pharmacodynamics and pricing of drugs (each *n* = 63, 1.7%).

**Table 1:** Characteristics of the responding physicians, presented as *n* (percentage)

		All (*n* = 3814)	Active in primary care (*n* = 609)	Active in other specialties (*n* = 3205)
		*n* (%)	*n* (%)	*n* (%)
Region	Västerbotten	416 (11)	84 (14)	332 (10)
	Västernorrland	305 (8.0)	67 (11)	238 (7.4)
	Östergötland	554 (15)	43 (7.1)	511 (16)
	Jönköping	478 (13)	118 (19)	360 (11)
	Västra Götaland	2007 (53)	290 (48)	1717 (54)
	Unknown/other	34 (0.9)	6 (1.0)	28 (0.9)
Age, years	≤ 50	2666 (70)	442 (73)	2224 (70)
	> 50	1136 (30)	165 (27)	971 (30)
Sex	Female	1939 (51)	333 (55)	1606 (51)
	Male	1836 (48)	271 (45)	1565 (49)
Country of education	In Sweden	2717 (71)	404 (67)	2313 (73)
	Outside Sweden	1078 (28)	202 (33)	876 (27)
Professional status	Specialist physician	2370 (62)	292 (48)	2078 (65)
	Junior doctors	1422 (37)	314 (52)	1108 (32)
Extent of patient work	Every day	3236 (85)	544 (89)	2692 (85)
Employment	Public	3690 (97)	557 (93)	3133 (99)
	Private	70 (1.8)	45 (7.5)	25 (0.8)
Research experience	No PhD	3047 (80)	557 (93)	2490 (78)
	PhD degree	736 (19)	44 (7.3)	692 (22)
Experience of work in a regional drug and therapeutics committee		686 (18)	108 (18)	578 (18)

**Table 2:** Drug-related queries at issue when searching for information as well as sources used (at least weekly) and factors perceived as important for the choice of drug information sources. Values are presented as numbers (percentage)

		All (*n* = 3814)	Active in primary care (*n* = 609)	Active in other specialties (*n* = 3205)
		*n* (%)	*n* (%)	*n* (%)
Drug related issue	Pharmacotherapy (e.g., dosing)	2888 (76)	567 (93)	2321 (72)
	Adverse effects	2384 (63)	531 (87)	1853 (58)
	Choice of drug	1819 (48)	456 (75)	1363 (43)
	Drug interactions	1724 (45)	407 (67)	1317 (41)
	Pharmacokinetics	1145 (30)	215 (35)	930 (29)
	Pregnancy	457 (12)	135 (22)	322 (10)
	Breast feeding	237 (7.2)	79 (13)	194 (6.1)
Sources of drug information used	Pharmaceutical Specialties in Sweden	3586 (94)	598 (98)	2988 (93)
	National online knowledge compilations	2446 (64)	516 (85)	1930 (60)
	Ask a colleague	1700 (45)	279 (46)	1421 (44)
	Google	1261 (33)	234 (38)	1027 (32)
	Regional drug and therapeutics committee, including their prescribing guidelines	762 (20)	310 (51)	452 (14)
	Medical literature in print	712 (19)	128 (21)	584 (18)
	PubMed	509 (13)	27 (4.4)	482 (15)
	Webpages of specialist associations	408 (11)	11 (1.8)	397 (12)
	Information from courses and conferences	402 (11)	75 (12)	327 (10)
	Swedish Medical Products Agency	348 (9.1)	80 (13)	268 (8.4)
	Drug Information Centers	192 (5.0)	50 (8.2)	142 (4.4)
	Pharmacies	151 (4.0)	35 (5.7)	116 (3.6)
	Cochrane	148 (3.9)	15 (2.5)	133 (4.1)
	Wikipedia	126 (3.3)	11 (1.8)	115 (3.6)
	Pharmaceutical companies	103 (2.7)	17 (2.8)	86 (2.7)
Factors perceived as important	Credibility	3349 (88)	536 (88)	2813 (88)
	Easy access, online	3127 (82)	494 (81)	2633 (82)
	Familiarity	2919 (77)	488 (80)	2431 (76)
	Update frequency	2341 (61)	404 (66)	1937 (60)
	Knowledge of origin/author	2087 (55)	349 (57)	1738 (54)
	Recommended by a colleague	1147 (30)	178 (29)	969 (30)
	Easy access, mobile application	451 (12)	62 (10)	389 (12)
	Available in print	123 (3.2)	29 (4.8)	94 (2.9)

The Pharmaceutical Specialties Inc. in Sweden was the source of drug information that almost all physicians reported using frequently (94%), followed by national online knowledge compilations (64%) (Table 2). Pharmaceutical Specialties in Sweden is integrated into all Swedish healthcare systems and contain electronic product information, including a Summary of Product Characteristics. In addition, among physicians in primary care, the regional drug and therapeutics committee, including regional prescribing guidelines, was a frequently used source of drug information (51%). Given the opportunity to list other sources of drug information not predefined in the questionnaire, 97 respondents (2.5%) reported that they frequently use the web-based decision support source UpToDate. Credibility was the most important factor for the choice of sources of drug information for 88% of the physicians, followed by easy access online, selected by 80% of physicians (Table 2).

Antibiotics were the group of drugs most often at issue in search of information (84%) (Table 3). For all drug groups except for antiepileptics and anticancer drugs, a larger proportion of physicians working in primary care stated that they often sought information compared with the entire group of physicians.

**Table 3:** Drug groups that physicians report to have searched for information about within the last 6 months. Reported as number (percentage)

	All (*n* = 3814)	Active in primary care (*n* = 609)	Active in other specialties (*n* = 3205)
	*n* (%)	*n* (%)	*n* (%)
Antibiotics	3215 (84)	569 (93)	2646 (83)
Analgesics	2657 (70)	512 (84)	2145 (67)
Anticoagulants	2576 (68)	492 (81)	2084 (65)
Cardiovascular drugs	2270 (60)	547 (90)	1723 (54)
Antidepressants	2261 (59)	568 (93)	1693 (53)
Antihypertensives	2153 (56)	509 (84)	1644 (51)
Antidiabetics	1868 (49)	550 (90)	1318 (41)
Antiepileptic drugs	1783 (47)	263 (43)	1520 (47)
Asthma/allergy drugs	1698 (45)	509 (84)	1189 (37)
Hypnotics	1606 (42)	409 (67)	1197 (37)
Antipsychotics	1559 (41)	270 (44)	1289 (40)
Gastrointestinal drugs	1487 (39)	390 (64)	1097 (34)
Dermatological drugs	1126 (30)	433 (71)	693 (22)
Antirheumatic drugs	1005 (26)	204 (33)	801 (25)
Anticancer drugs	926 (24)	84 (14)	842 (26)
Parkinson drugs	814 (21)	208 (34)	606 (19)
Gynecological drugs	638 (17)	230 (38)	408 (13)
Other drugs	453 (12)	31 (5.1)	422 (13)
Herbal drugs	362 (9.5)	105 (17)	257 (8.0)

The questionnaire also contained questions concerning the type and provider of oral drug information at the physicians’ workplace. Overall, the physicians responded that the information most frequently concerned a specific drug (*n* = 2081, 65%). For physicians working in primary care, the corresponding number was lower, *n* = 274 (45%). Regarding the provider of drug information, a total of 2641 (69%) physicians responded that the information usually was given by representatives from pharmaceutical companies. For physicians in primary care, the corresponding number was 321 (53%). A total of 244 (6.4%) physicians and 154 (25%) physicians working in primary care, reported that the drug information mostly was provided by representatives from the public regions. About a quarter of the respondents reported that they never received any drug information at all at their workplace; *n* = 997 (26%) for the whole group of physicians and *n* = 146 (24%) for physicians working in primary care.

In a more detailed analysis among physicians working in primary care, several responder characteristics were associated with usage of, as well as preferences of, drug information sources (Table 4). Overall juniority, i.e., status as a junior doctor and age 50 years and younger, was associated with more frequent use of drug information. The significant difference between men and women regarding the usage of information about drugs in pregnancy remained even after division by professional status.

**Table 4:** Statistically significant associations between responder characteristics and replies (answers options) among physicians working in primary health care

Personal factors	Answer options	Question about*	***P***-values**	Comparison (%)	
Age				≤ 50 years	> 50 years
	Ask a colleague	Source of drug information	< 0.0001	56	19
	Easy access, online	Important factor	< 0.0001	90	74
	Pharmacotherapy (e.g., dosing)	Drug-related issue	< 0.0001	96	86
	Recommended by a colleague	Important factor	0.0021	34	17
	Adverse effects	Drug-related issue	0.016	90	79
Professional status				Junior doctors	Specialists
	Ask a colleague	Source of drug information	< 0.0001	64	26
	Recommended by a colleague	Important factor	< 0.0001	40	18
Research experience				PhD	No PhD
	PubMed	Source of drug information	< 0.0001	18	3.2
Sex				Men	Women
	Pregnancy	Drug-related issue	0.004	30	16
	Recommended by a colleague	Important factor	0.037	21	36
	Ask a colleague	Source of drug information	0.042	38	52

*“Drug related issue” refers to Table 2 Section 1, “Source of drug information” refers to Table 2 Section 2, “Important factor” refers to Table 2 Section 3, and “Group of drugs” refers to Table 3

**After Bonferroni correction. Table 4 is also available as an online supplemental table which contains more extensive results

## Discussion

The present study, covering five regions in Sweden and, to the best of our knowledge, the largest study hitherto performed, confirms physicians’ need for drug information. It appears that physicians use multiple sources in the search for several drug-related issues, including, for example, dosing, adverse drug reactions, drug choices, and drug interactions. Furthermore, credibility, easy online access, familiarity, update frequency, and knowledge of origin or author seem to be crucial for their choice of drug information source. In addition, we found several personal factors associated with physicians’ choice of information sources.

Most of the physicians in our study searched for information about drugs at least weekly. Since drug dosing errors [5] and adverse drug reactions [6, 7] both are frequently occurring in health care, the finding of pharmacotherapy including dosing as the most common drug-related issue for searching and adverse effects as the next most common may be reassuring.

There was a similar pattern for physicians in primary care as for physicians active in other specialties with respect to the most commonly used sources for drug information, i.e., Pharmaceutical Specialties Inc. in Sweden as well as national online knowledge compilations. Our result that colleagues are an important source of information is consistent with two prior studies, both published in 2013 [8, 9]. However, asking a colleague was considerably less common in a study from Ireland, published in 2001, where 7% of general practitioners (GPs) and 29% of hospital doctors consulted colleagues for prescribing information [4].

In our study, the most conspicuous difference in the usage of sources of information between physicians active in primary care and physicians active in other specialties was that the former used information from the regional drug and therapeutic committee, e.g., regional prescribing guidelines, to a greater extent and PubMed and webpages of specialist associations to a smaller extent. These results may not be surprising. Indeed, regional prescribing guidelines are intended for physicians in primary care. A previous questionnaire study among 603 Swedish physicians in 2008 in primary care revealed that 97% used these guidelines [10], and in general, specialist associations do not focus on primary care.

Notably, about one-third of all physicians frequently use the search engine Google when searching for drug information. This is consistent with results from other studies that report high use of non-authoritative sources, including Google [11–13]. A descriptive survey including 444 randomly selected physicians in Nigeria, published in 2011, reported Google being the most frequently used search engine (73%), and PubMed being the most used medical database (70%) [11]. In a study from 2015 among medical residents in New Jersey, 56% reported using Google on mobile devices. However, that study reported that Google was primarily used to identify foreign drugs and only by 2.7% for questions regarding dosing [12]. The use of non-authoritative online information sources such as Google implies that the prescriber needs to be able to differentiate between reliable and non-reliable sources. In this context, it should be noted that a minority of our respondents had a PhD degree, and furthermore, a majority had no research experience at all. Nevertheless, 88% stated that credibility was the most important factor when choosing sources of drug information. This may imply that they find it important to evaluate reliability of information sources. This is consistent with a systematic review where credibility was one of five listed factors that could have significant effects on physicians’ choices of information sources [1].

In our study, there was a difference in who provides doctors with oral drug information at work. In the previously mentioned Irish study, the GPs reported consulting the pharmaceutical industry to a greater extent (42%) than the hospital doctors (18%) before prescribing a new drug [4]. In another previous Swedish study from 2011, about 85% of the GPs responding to a questionnaire reported getting too much information from the pharmaceutical industry [14]. In contrast, physicians in primary care in our study stated to a greater extent that the information was provided by representatives employed in the regions, while physicians in specialist care stated that the information came to a greater extent from the pharmaceutical companies. This could have implications for prescribing since a previous study published in 2010 showed that the presence of drug information from the pharmaceutical industry was negatively associated with adherence to the prescribing objectives [15]. It was also notable that about one-quarter of our respondents stated that they do not receive any information at all about drugs at their workplace, since prescribing drugs is an important professional activity for physicians that requires continuous updating.

In a study from Ethiopia published in 2013, respondents from a specialized hospital were more likely to consult drug information sources compared with respondents from primary care [16]. In our study, the relationship was reversed. Of physicians in primary care, 96% reported searching for information about drugs at least every week, compared with 83% for physicians active in specialties others than primary health care. This difference between our study and the previous one may reflect the extensive availability of drug information in primary care in a developed country compared to a developing country. Indeed, most of our respondents from primary care had searched for information over the last 6 months for the majority of the predefined groups of drugs. This seems logical since physicians in primary care meet patients with varying diseases, including older people with multiple morbidities.

Among physicians in primary care, we found that professional status as a junior doctor, as well as younger age, was associated with more frequent use of drug information in general. This finding is in line with a study from 2012 in which 721 French GPs answered a questionnaire about using the Internet for clinical information [17]. In addition, professional status as a junior doctor and younger age were, in our study, also associated with advice taken from colleagues. Interestingly, female primary care physicians more often used colleagues as a source of information and also found sources recommended by colleagues more reliable. This result is consistent with a previous study from Denmark, published in 2016, where young and female GPs were more likely to seek information from colleagues [18]. Unexpectedly, male physicians in primary care sought a greater extent of information about drugs in pregnancy compared with their female counterparts. Finally, our results of more extensive use of PubMed for physicians in primary care with research experience seem reasonable.

A large number of respondents is a strength of this study. However, the low response rate is an apparent limitation, affecting the generalizability of the results. In general, however, the response rate tends to be low in questionnaire studies of physicians [19]. To maximize the response rate, we kept the number of questions limited, used predefined answers, and sent several reminders. On the other hand, predefined multiple-choice answers may be considered a limitation. Although they were phrased by knowledgeable researchers, it cannot be excluded that relevant aspects were left out. The free-text response options may, to some extent, compensate for this issue, but the threshold to respond to such questions is probably higher. Although performed in five Swedish regions, it may also be regarded as a limitation that our study was restricted to Sweden. Indeed, international comparisons could add valuable insights.

## Conclusion

This study confirms that physicians often use drug information sources in professional work, in particular those who work in primary health care. Credibility and easy access online were key factors for usage, and pharmacotherapy and adverse drug reactions were the most common issues. For physicians in primary care, personal factors, such as juniority and sex, were associated with usage and preferences of drug information sources.

## Supplementary Information

Below is the link to the electronic supplementary material.

Supplementary file1 (DOCX 56 KB)

## Data Availability

Available upon request from the first author.
