# Supplementary material for: Drug information sources in professional work—a questionnaire study on physicians’ usage and preferences (the drug information study)
Source: Eur J Clin Pharmacol. 2023 Apr 14;79(6):767–74. doi: 10.1007/s00228-023-03494-4 (PMC10229724; doi:10.1007/s00228-023-03494-4)
Supplement: Supplementary file 1 — Supplementary file1 (DOCX 56 KB) [file 228_2023_3494_MOESM1_ESM.docx]

**Table 4** Statistically significant associations between responder characteristics and replies (answers options) among physicians working in primary health care.

| Personal factors | Answer options | Question about *) | Non-corrected p-values | P-values **) | Comparison (%) | |
| --- | --- | --- | --- | --- | --- | --- |
|  |  |  |  |  |  |  |
| Age |  |  |  |  | ≤50 years | >50 years |
|  | Ask a colleague | Source of drug information | 2.2E-16 | 1.1E-14 | 56 | 19 |
|  | Antihypertensive | Group of drugs | 1.2E-07 | 5.7E-06 | 89 | 71 |
|  | Asthma / allergy drugs | Group of drugs | 1.9E-07 | 9.1E-06 | 88 | 71 |
|  | Gastrointestinal drugs | Group of drugs | 9.8E-07 | 4.7E-05 | 70 | 48 |
|  | Insomnia | Group of drugs | 9.6E-07 | 4.6E-05 | 73 | 52 |
|  | National online knowledge compilation | Source of drug information | 6.5E-07 | 3.1E-05 | 90 | 74 |
|  | Dermatological drugs | Group of drugs | 6.2E-07 | 3E-05 | 77 | 56 |
|  | Pharmacotherapy (e.g. dosing) | Drug related issue | 1.6E-05 | 7.8E-04 | 96 | 86 |
|  | Recommended by a colleague | Important factor | 4.4E-05 | 0.0021 | 34 | 17 |
|  | Adverse effects | Drug related issue | 3.4E-04 | 0.016 | 90 | 79 |
|  | Antirheumatics | Group of drugs | 0.001 | NS | 37 | 24 |
|  | Herbal remedies | Group of drugs | 0.002 | NS | 20 | 10 |
|  | Pharmaceutical Specialties in Sweden | Source of drug information | 0.002 | NS | 99 | 96 |
|  | Analgesic | Group of drugs | 0.005 | NS | 87 | 78 |
|  | Gynecological drugs | Group of drugs | 0.006 | NS | 41 | 29 |
|  | Information from courses and conferences | Source of drug information | 0.009 | NS | 14 | 6.7 |
|  | Antidepressants | Group of drugs | 0.024 | NS | 95 | 90 |
|  | Antibiotics | Group of drugs | 0.017 | NS | 95 | 90 |
|  |  |  |  |  |  |  |
|  |  |  |  |  |  |  |
| Country of ed. |  |  |  |  | Sweden | Outside Sweden |
|  | Regional drug and therapeutics committee, including their prescribing guidelines | Source of drug information | 3.8E-10 | 1.8E-08 | 60 | 33 |
|  | Credibility | Important factor | 1E-07 | 4.8E-06 | 93 | 78 |
|  | Medical literature in print | Source of drug information | 2.7E-08 | 1.3E-06 | 15 | 34 |
|  | Knowledge of origin/author | Important factor | 7.8E-06 | 3.7E-04 | 64 | 45 |
|  | Pregnancy | Drug related issue | 6E-05 | 0.0029 | 17 | 40 |
|  | Familiarity | Important factor | 0.001 | NS | 84 | 73 |
|  | Wikipedia | Source of drug information | 0.001 | NS | 0.5 | 4.5 |
|  | Choice of drug | Drug related issue | 0.002 | NS | 79 | 67 |
|  | Breastfeeding | Drug related issue | 0.003 | NS | 10 | 19 |
|  | Information from courses and conferences | Source of drug information | 0.004 | NS | 10 | 18 |
|  | Easy access, mobile application | Important factor | 0.008 | NS | 7.9 | 15 |
|  | PubMed | Source of drug information | 0.012 | NS | 3.0 | 7.4 |
|  | Easy access, online | Important factor | 0.012 | NS | 84 | 76 |
|  | Update frequency | Important factor | 0.015 | NS | 63 | 73 |
|  | Ask a colleague | Source of drug information | 0.021 | NS | 49 | 39 |
|  | Available in print | Important factor | 0.031 | NS | 3.5 | 7.4 |
|  | Antidiabetic drugs | Group of drugs | 0.043 | NS | 92 | 87 |
|  | Neuroleptics | Group of drugs | 0.043 | NS | 47 | 39 |
|  |  |  |  |  |  |  |
|  |  |  |  |  |  |  |
| Professional position |  |  |  |  | Junior doctors | Specialists |
|  | Ask a colleague | Source of drug information | 1.5E-20 | 7.2E-19 | 64 | 26 |
|  | Recommended by a colleague | Important factor | 4.9E-09 | 2.4E-07 | 40 | 18 |
|  | Asthma / allergy drugs | Group of drugs | 1.8E-07 | 8.5E-05 | 90 | 76 |
|  | Antihypertensives | Group of drugs | 8.1E-06 | 3.9E-04 | 90 | 77 |
|  | Dermatological drugs | Group of drugs | 3.2E-05 | 0.002 | 79 | 63 |
|  | Insomnia | Group of drugs | 1.8E-04 | 0.009 | 74 | 60 |
|  | Pharmacotherapy (e.g. dosing) | Drug related issue | 0.002 | NS | 96 | 90 |
|  | Pharmacy | Drug related issue | 0.007 | NS | 3.2 | 8.2 |
|  | Webpages of specialist associations | Source of drug information | 0.009 | NS | 3.2 | 0.3 |
|  | Antirheumatics | Group of drugs | 0.014 | NS | 38 | 29 |
|  | Gastrointestinal drugs | Group of drugs | 0.014 | NS | 69 | 59 |
|  | Knowledge of origin/author | Important factor | 0.015 | NS | 52 | 62 |
|  | Familiarity | Important factor | 0.017 | NS | 84 | 76 |
|  | Update frequency | Important factor | 0.029 | NS | 70 | 62 |
|  | Gynecological drugs | Group of drugs | 0.032 | NS | 42 | 34 |
|  | Regional drug and therapeutics committee, including their prescribing guidelines | Source of drug information | 0.033 | NS | 47 | 55 |
|  | Parkinson disease drugs | Group of drugs | 0.036 | NS | 30 | 38 |
|  |  |  |  |  |  |  |
| Public/private |  |  |  |  | Public | Private |
|  | Ask a colleague | Source of drug information | 0.019 | NS | 47 | 29 |
|  | Recommended by a colleague | Important factor | 0.042 | NS | 30 | 16 |
|  |  |  |  |  |  |  |
| Research experience |  |  |  |  | No PhD | PhD |
|  | PubMed | Source of drug information | 2.7E-06 | 1.3E-04 | 3.2 | 18 |
|  | Adverse effects | Drug related issue | 0.046 | 0.057 | 87 | 77 |
|  | Pharmacotherapy (e.g. dosing) | Drug related issue | 0.016 | NS | 93 | 84 |
|  | Familiarity | Important factor | 0.038 | NS | 83 | 68 |
|  |  |  |  |  |  |  |
| Sex |  |  |  |  | Men | Women |
|  | Pregnancy | Drug related issue | 9.1E-05 | 0.004 | 30 | 16 |
|  | Recommended by a colleague | Important factor | 7.7E-05 | 0.037 | 21 | 36 |
|  | Ask a colleague | Source of drug information | 0.001 | 0.042 | 38 | 52 |
|  | Easy access, online | Important factor | 0.013 | NS | 77 | 85 |
|  | Credibility | Important factor | 0.02 | NS | 85 | 88 |
|  | Antidepressants | Group of drugs | 0.022 | NS | 96 | 91 |
|  | Antiepileptics | Group of drugs | 0.033 | NS | 48 | 39 |
|  | Familiarity | Important factor | 0.048 | NS | 77 | 83 |

*) “Drug related issue” refers to table 2 section 1, “Source of drug information” refers to table 2 section 2, “Important factor” refers to table 2 section 3, and “Group of drugs” refers to table 3.

**) After Bonferroni correction.
